# The serotonin receptor 3E variant is a risk factor for female IBS-D

**DOI:** 10.1007/s00109-022-02244-w

**Published:** 2022-09-19

**Authors:** Nikola Fritz, Sabrina Berens, Yuanjun Dong, Cristina Martínez, Stefanie Schmitteckert, Lesley A. Houghton, Miriam Goebel-Stengel, Verena Wahl, Maria Kabisch, Dorothea Götze, Mauro D’Amato, Tenghao Zheng, Ralph Röth, Hubert Mönnikes, Jonas Tesarz, Felicitas Engel, Annika Gauss, Martin Raithel, Viola Andresen, Jutta Keller, Thomas Frieling, Christian Pehl, Christoph Stein-Thöringer, Gerard Clarke, Paul J. Kennedy, John F. Cryan, Timothy G. Dinan, Eamonn M. M. Quigley, Robin Spiller, Caroll Beltrán, Ana María Madrid, Verónica Torres, Emeran A. Mayer, Gregory Sayuk, Maria Gazouli, George Karamanolis, Mariona Bustamante, Xavier Estivil, Raquel Rabionet, Per Hoffmann, Markus M. Nöthen, Stefanie Heilmann-Heimbach, Börge Schmidt, André Franke, Wolfgang Lieb, Wolfgang Herzog, Guy Boeckxstaens, Mira M. Wouters, Magnus Simrén, Gudrun A. Rappold, Maria Vicario, Javier Santos, Rainer Schaefert, Justo Lorenzo-Bermejo, Beate Niesler

**Affiliations:** 1 Institute of Human Genetics, Department of Human Molecular Genetics, Heidelberg University Hospital, Heidelberg, Germany; 2 Department of General Internal Medicine and Psychosomatics, Heidelberg University Hospital, Heidelberg, Germany; 3 Institut de Recerca Biomèdica de Lleida (IRBLleida), Lleida, Spain; 4 Lleida Institute for Biomedical Research Dr, Pifarré Foundation (IRBLleida), Lleida, Spain; 5 University of Leeds, St. James’s University Hospital, Leeds, UK; 6 Mayo Clinic, Jacksonville, FL USA; 7 Department of Psychosomatic Medicine, University Hospital Tübingen, Tübingen, Germany; 8 Department of Internal Medicine and Gastroenterology, HELIOS Clinic Rottweil, Rottweil, Germany; 9 Institute of Medical Biometry and Informatics, Heidelberg University, Heidelberg, Germany; 10 Unit of Clinical Epidemiology, Department of Medicine Solna, Karolinska Institutet, Stockholm, Sweden; 11 Gastrointestinal Genetics Lab, CIC bioGUNE - BRTA, Bilbao, Derio Spain; 12 IKERBASQUE, Basque Foundation for Science, Bilbao, Spain; 13 nCounter Core Facility, Department of Human Molecular Genetics, Heidelberg University Hospital, Heidelberg, Germany; 14 Martin-Luther-Hospital, Berlin, Germany; 15 Department of Gastroenterology, Infectious Diseases and Intoxications, Heidelberg University, Heidelberg, Germany; 16 University of Erlangen, Erlangen, Germany; 17 Israelitisches Krankenhaus, Hamburg, Germany; 18 Helios Klinik Krefeld, Krefeld, Germany; 19 Krankenhaus Vilsbiburg, Vilsbiburg, Germany; 20 German Cancer Research Center, Heidelberg, Germany; 21 Department of Psychiatry and Neurobehavioral Science, University College Cork, Cork, Ireland; 22 APC Microbiome Ireland, University College Cork, Cork, Ireland; 23 Department of Anatomy and Neuroscience, University College Cork, Cork, Ireland; 24 Lynda K. and David M. Underwood Center for Digestive Disorders, Houston Methodist Hospital, Weill Cornell Medical College, Houston, TX USA; 25 Nottingham Digestive Diseases Centre, University of Nottingham, Nottingham, UK; 26 Gastroenterology Unit, Medicine Department, Hospital Clínico Universidad de Chile, Universidad de Chile, Santiago de Chile, Chile; 27 Oppenheimer Center for Neurobiology of Stress, University of California, Los Angeles, CA USA; 28 Washington University School of Medicine, St. Louis, MO USA; 29 Laboratory of Biology, Medical School, National and Kapodistrian University of Athens, Athens, Greece; 30 Academic Department of Gastroenterology, Medical School, National and Kapodistrian University of Athens, Laikon General Hospital, Athens, Greece; 31 CRG, Centre for Genomic Regulation, Barcelona, Spain; 32 ISGlobal, Barcelona, Spain; 33 Department of Genetics, Microbiology and Statistics, Faculty of Biology, IBUB, Universitat de Barcelona, CIBERER, IRSJD, Barcelona, Spain; 34 Life and Brain Center, Bonn, Germany; 35 Institute for Medical Informatics, Biometry and Epidemiology, University Hospital of Essen, Essen, Germany; 36 Institute of Clinical Molecular Biology, Kiel, Germany; 37 Institute of Epidemiology, Kiel, Germany; 38 TARGID, University Hospital Leuven, Louvain, Belgium; 39 Institute of Medicine, University of Gothenburg, Gothenburg, Sweden; 40 Interdisciplinary Center for Neurosciences (IZN), Heidelberg University, Heidelberg, Germany; 41 Institut de Recerca Vall d Hebron, Hospital Vall d Hebron, Passeig de la Vall d Hebron, Barcelona, Spain; 42 Nestlé Institute of Health Sciences, Nestlé Research, Société Des Produits Nestlé S.A, Vers-chez-les-Blanc, Lausanne, Switzerland; 43 Department of Psychosomatic Medicine, Division of Theragnostics, University Hospital Basel, Basel, Switzerland; 44 Faculty of Medicine, University of Basel, Basel, Switzerland

**Keywords:** Irritable bowel syndrome, Serotonin type 3 receptor, IBS-D, Females

## Abstract

Irritable bowel syndrome (IBS) is a gut-brain disorder of multifactorial origin. Evidence of disturbed serotonergic function in IBS accumulated for the 5-HT_3_ receptor family. 5-HT_3_Rs are encoded by *HTR3* genes and control GI function, and peristalsis and secretion, in particular. Moreover, 5-HT_3_R antagonists are beneficial in the treatment of diarrhea predominant IBS (IBS-D). We previously reported on functionally relevant SNPs in *HTR3A* c.-42C > T (rs1062613), *HTR3C* p.N163K (rs6766410), and *HTR3E* c.*76G > A (rs56109847 = rs62625044) being associated with IBS-D, and the *HTR3B* variant p.Y129S (rs1176744) was also described within the context of IBS. We performed a multi-center study to validate previous results and provide further evidence for the relevance of *HTR3* genes in IBS pathogenesis. Therefore, genotype data of 2682 IBS patients and 9650 controls from 14 cohorts (Chile, Germany (2), Greece, Ireland, Spain, Sweden (2), the UK (3), and the USA (3)) were taken into account. Subsequent meta-analysis confirmed *HTR3E* c.*76G > A (rs56109847 = rs62625044) to be associated with female IBS-D (OR = 1.58; 95% CI (1.18, 2.12)). Complementary expression studies of four GI regions (jejunum, ileum, colon, sigmoid colon) of 66 IBS patients and 42 controls revealed only *HTR3E* to be robustly expressed. On top, *HTR3E* transcript levels were significantly reduced in the sigma of IBS patients (*p* = 0.0187); more specifically, in those diagnosed with IBS-D (*p* = 0.0145). In conclusion, meta-analysis confirmed rs56109847 = rs62625044 as a risk factor for female IBS-D. Expression analysis revealed reduced *HTR3E* levels in the sigmoid colon of IBS-D patients, which underlines the relevance of *HTR3E* in the pathogenesis of IBS-D.

**Supplementary Information:**

The online version contains supplementary material available at 10.1007/s00109-022-02244-w.

## Introduction

The paradigm of the chronic gastrointestinal (GI) disorder irritable bowel syndrome (IBS) as ‘*functional*’ disorder recently shifted to that of a ‘*prototypical gut*-*brain disorder*’ [1]. Patients suffer from abdominal pain in conjunction with altered bowel habits such as diarrhea, constipation, mixed/alternating, or unspecified pattern: IBS-D, IBS-C, IBS-M/IBS-A, or IBS-U, respectively [2, 3]. IBS represents one of the common GI disorders depending on the Rome criterion employed; a prevalence has ranged from 10.1% (Rome III) to 4.1% (Rome IV) worldwide [4] and 70–75% of the affected individuals are female [2, 5].

The immune system, hormones, and neurotransmitters have a major influence on the impaired bidirectional communication via the gut-brain axis [2, 3]. Among these, serotonin (5-HT, 5-hydroxytryptamine) is a key regulator acting polyfunctionally as neurotransmitter, paracrine factor, endocrine hormone, as well as growth factor [6]. Intestinal 5-HT regulates various GI functions including motility, secretion, as well as visceral sensation [6]. Of note, alterations in both central and peripheral 5-HT systems contribute to visceral hypersensitivity in IBS [7]. Furthermore, 5-HT is well-known to influence behavior and modulate the immune and nervous systems, through affecting the vagus nerve. 5-HT has also been shown to be relevant to GI and comorbid disorders [6, 8, 9]. More than 90% of the body’s serotonin is actually produced in the gut where it is influenced by gut microbiota [10].

Extrinsic factors such as stress, infection, and nutrition in concert with intrinsic factors including the individual genetic background and microbiota have been proven to lead to a predisposition to IBS [7, 11].

Evidence of disturbed serotonergic function in IBS has accumulated for the serotonin type 3 (5-HT_3_) receptor family [11, 12]. Functional 5-HT_3_ receptors are composed of five subunits (5-HT3A-E) encoded by the genes *HTR3A*, *HTR3B*, *HTR3C*, *HTR3D*, and *HTR3E* [13]. They build ligand-gated ion channels of diversely composed pentameric complexes [13].

5-HT_3_ receptors control GI function, in particular, peristalsis and secretion and 5-HT_3_R antagonists have been proven to be beneficial in the treatment of IBS-D as well as in comorbid psychiatric conditions [13]. Furthermore, they are relevant in emotional processing, mood and visceral perception and have been associated with anxiety and depression, disorders that are frequently comorbid with IBS [13]. Several case control and pharmacogenetic studies revealed an association between *HTR3* variants and both, psychiatric and neurogastroenterologic phenotypes. Recently, findings of associations in various conditions as well as their potentials as predictors of pharmacoresponse have been collected in the serotonin receptor type 3 *HTR3* gene allelic variant database (www.htr3.uni-hd.de) [12] containing five sub-databases. The data comprise *HTR3* variants, their functional relevance, associated phenotypes, and pharmacogenetic data of the five different serotonin receptor genes *HTR3A*-*E*.

We previously reported that single-nucleotide polymorphisms (SNPs) in *HTR3A* c.-42C > T (rs1062613), *HTR3C* p.N163K (rs6766410), and *HTR3E* c.*76G > A (rs56109847 = rs62625044) were associated with IBS-D [14, 15]. More specifically, the SNPs in *HTR3C* and *HTR3E* were associated with female IBS-D [14, 15]. The *HTR3A* and *HTR3E* SNPs represent *cis*-regulatory variants. The c.-42C > T SNP is located within the 5′UTR of *HTR3A*, and c.*76G > A within the 3′UTR of *HTR3E* in a microRNA miR-510 binding site, respectively [14]. Both SNPs seem to impair expression regulation and cause upregulation of receptor expression. Lately, their association with IBS-D was replicated in four Chinese studies and recent genome-wide association studies showed a trend for *HTR3A* and *HTR3E* with IBS [16–21]. p.Y129S in HTR3B (rs1176744) that we had previously found to be associated with anorexia and depression was also reported in the context of IBS in a Japanese study, and was noted to be particularly associated with increased anxiety scores and alexithymia [22–24]. Some additional evidence for the role of HTR3 variants arose from genetic imaging studies [11].

Our hypothesis is that the predisposing SNPs in *HTR3A*, *HTR3B*, *HTR3C*, and *HTR3E* encode 5-HT3 receptor subunits of disturbed structure and impaired function, thereby making people prone to develop IBS.

**Fig. 1:**
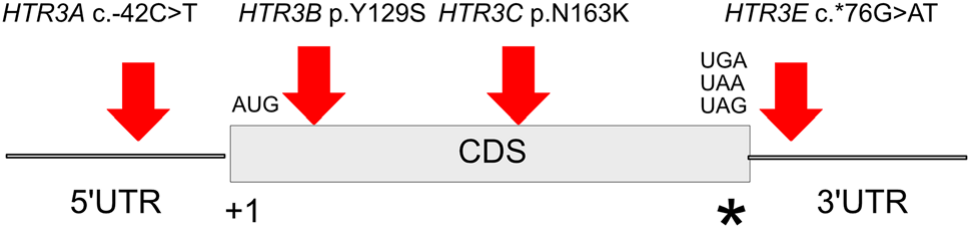
Schematic of the *HTR3* genes indicating the location and type of the analyzed SNPs. The gray box indicates the coding sequence of a *HTR3* gene. The upstream region of the start codon AUG (5′untranslated region [5′UTR]) is indicated by the thin line on the left, whereas the respective 3′ untranslated region resides downstream of the stop codon, illustrated by an asterisk on the right. CDS, coding sequence (protein coding portion)

In this study, we aimed at unraveling the impact of the *HTR3* SNPs on IBS pathogenesis which had previously been reported to associate with IBS. Joining efforts of the German IBS research network and the European network GENIEUR (The Genes in Irritable Bowel Syndrome Research Network Europe; www.GENIEUR.eu) enabled us to perform a multi-center study to validate previous association findings of functional SNPs of the serotonin type 3 receptor subunit genes *HTR3A* c.-42C > T (rs1062613), *HTR3B* p.Tyr129Ser (rs1176744), *HTR3C* p.N163K (rs6766410), and *HTR3E* c.*76G > A (rs56109847 = rs62625044) (illustrated in Fig. 1) and provide further evidence for the relevance of serotonin type 3 receptors to the aetiology of IBS. In order to validate initial association findings, we genotyped the SNPs in 13 additional cohorts from eight countries including Chile, Germany, Greece, Ireland, Spain, Sweden, the UK, and the USA. Meta-analysis revealed a replication of the initial finding for *HTR3E* only. Functional follow-up of associated variants was performed by complementing comparative expression analysis in different GI regions including jejunum, ileum and colon, respectively. Finally, genotype–phenotype correlation was performed to allow an insight into functional consequences for SNP carriers.

## Material and methods

The experimental design is summarized in Supplementary SD Fig. 1.

### IBS patients and healthy controls

In addition to the discovery cohort from the UK, SNP analysis was carried out on DNAs of 2485 IBS patients of expert centers and 9560 control individuals of expert centers from 13 cohorts from eight countries including Chile, Germany, Greece, Ireland, Spain, Sweden, the UK, and the USA. The diagnosis of IBS and bowel habit subtyping was determined based on either Rome II or Rome III criteria (see Supplementary SD Table 1). The following expert centers included study individuals: IBS-Net Germany: IBS outpatient clinic at the University Hospital Heidelberg; Israelitisches Krankenhaus, Hamburg; Helios Klinikum Krefeld, Krefeld; Martin-Luther-Krankenhaus, Berlin; Krankenhaus Vilsbiburg, Vilsbiburg; Klinikum rechts der Isar, Munich; all from Germany. Furthermore, Hospital Clínico Universidad de Chile, Santiago de Chile, Chile; Aretaieion Hospital and ‘Laikon’ General Hospital, Athens, Greece; Alimentary Pharmabiotic Centre, University College Cork, Cork, Ireland; Hospital Universitari Vall d´Hebron, Barcelona, Spain; IBS-Net Sweden with the following centers: Karolinska University Hospital Stockholm, Stockholm; Department of Medicine, Umeå University, Umeå, Sweden; Division of Gastroenterology, Institution of Clinical and Experimental Medicine, Linköping University, Linköping; Department of Clinical Sciences, Skånes University Hospital, Malmoe; Department of Internal Medicine & Clinical Nutrition, Institute of Medicine, Sahlgrenska Academy, University of Gothenburg, Gothenburg, all in Sweden; Neurogastroenterology Unit, University of Manchester, Wythenshawe Hospital, Manchester, UK. Furthermore, patients were enrolled in a clinical trial (identifier NCT00745004 at clinicaltrials.gov) at the NIHR Nottingham Biomedical Research Centre, Nottingham University Hospitals, NHS Trust and the University of Nottingham, Nottingham, UK; Division of Gastroenterology, John T. Milliken, Department of Medicine, Washington University School of Medicine, St.Louis, WA, USA; Oppenheimer Center for the Neurobiology of Stress, Division of Digestive Diseases Los Angeles, CA, USA. Moreover, a cohort of IBS patients and controls from the UK, USA, and Canada was included kindly provided by Glaxo Smith Kline.

**Table 1:** Meta-analysis Data *HTR3E* rs56109847 = rs62625044 (IBS-D females), the discovery cohort UK1 is indicated in bold letters

Cohort	OR 95%	Lower	Upper
Germany1	4.92	1.49	16.30
Germany2	2.17	0.76	6.17
Greece	1.85	0.41	8.32
Spain	1.31	0.39	4.49
Sweden1	1.00	0.23	4.29
Sweden2	1.32	0.71	2.45
**UK1**	**8.55**	**1.04**	**70.50**
UK2	2.53	0.67	9.61
UK3	2.91	1.14	7.44
USA1	0.39	0.05	3.29
USA2	1.08	0.58	2.03
USA3	0.17	0.02	1.66
**Mantel–Haenszel OR**	**1.58**	**1.18**	**2.12**

Furthermore, control data from the Heinz-Nixdorf Recall (HNR) Study and the PopGen Health Study (both Germany), INMA (Spain), and SALT (Sweden) were also taken into account as well as DNAs that had been extracted from GI tissues and used for expression analyses.

Comparative expression analyses of *HTR3* genes were carried out on small and large intestinal biopsies of three case–control cohorts from Spain (JS, Barcelona) and Germany (MGS, Berlin and ME, Erlangen-Nürnberg) (see Supplementary SD Table 2).

All participants were of Caucasian ancestry. Written informed consent was obtained from all subjects and the experiments conformed to the principles of the WMA Declaration of Helsinki and the Department of Health and Human Services Belmont Report. All studies were approved by the local ethics committees, as outlined under Declarations in detail.

### Preparation of genomic DNA

Genomic DNA was prepared from blood or saliva samples taken from both patients and healthy controls using standard protocols, as described previously [14, 25] for all except the ones stated in the Supplementary data.

### KASP genotyping assay

SNP genotyping of the SNPs was carried out applying the KASPar® assay (KBiosciences Ltd., Hoddesdon, UK), as recommended by the manufacturer (for Primer sequences see Supplementary SD Table 3) for all except the ones stated in the Supplementary data. Thermal cycling was performed in Mastercycler *vapo.protect* thermal cyclers (Eppendorf). An initial 15 min incubation at 95 °C was followed by 20 cycles consisting of s at 94 °C, 5 s at 57 °C, and 10 s at 72 °C, followed by 23 cycles consisting of 10 s at 94 °C, 5 s at 57 °C, and 10 s at 72 °C. After thermal cycling, results were analyzed using the fluorescence plate reader of the 7500 Fast Real-Time PCR System (Applied Biosystems, Foster City, California). Ten percent of the samples were repeated for quality control constraints and could be confirmed.

### RNA extraction and reverse transcription

To quantify gene expression and to correlate differential expression driven by the *HTR3* SNPs, total RNA was extracted from small and large intestine tissues (jejunum, ileum, colon, sigmoid colon) of IBS patients and control individuals using TRIzol reagent (Thermo Fisher Scientific, Waltham, USA). One microgram of total RNA was reverse transcribed into complementary DNA (cDNA) using the Superscript III-First-Strand-Synthesis-System (Invitrogen), as recommended by the manufacturer.

### Quantitative PCR

Relative gene expression was analyzed by Quantitative PCR (qPCR) on a 7500 Fast Real-Time PCR System (Applied Biosystems, Foster City, California) using TaqMan Assays according to the manufacturer’s instructions (Applied Biosystems), as specified in Supplementary SD Table 4). All values were normalized to 18S RNA (Applied Biosystems, TaqMan Assay ID: Hs99999901_s1). Each sample, including sterile distilled water as a negative control, was run in triplicate and data were analyzed by the 2-∆∆Ct method with correction for primer efficiency.

### Statistical analysis

#### Statistical analysis of the genotyping data

Genotype frequencies, association analyses, and tests for deviation from the Hardy–Weinberg Equilibrium (HWE) were compared as described previously [14]. Genotype-relative risks of IBS and IBS subtypes were quantified by odds ratios (ORs) with the corresponding 95% confidence intervals (CIs) based on a logistic regression model under a dominant inheritance model (homozygous genotype major allele compared with hetero- and homozygous genotype minor allele), except for *HTR3C* in which the initial finding had been under a recessive inheritance model (homozygous genotype major allele andheterozygous major/minor allele compared to the homozygous genotype of the minor allele) [26].

#### Meta-analysis

Results from single studies were combined using fixed and random effects meta-analyses. Results were represented by Forest plots, as follows: CIs on the OR for each study were indicated by horizontal lines, study-specific ORs by squares proportional to the study size, and combined summary estimates by a diamond with horizontal limits indicating the confidence limits. Data were analyzed using the rmeta package from the free Software Environment for Statistical Computing R.

To address the impact of the study size on the outcome of the meta-analysis, funnel plots were generated.

## Results

Genoytpe data for *HTR3* SNPs of 2682 IBS patients and 9650 controls of 14 cohorts from Chile, Germany (2), Greece, Ireland, Spain, Sweden (2), the UK (3), and the USA (3) (Supplementary SD Table 5, Fig. 1) were considered to validate previous association findings with IBS. Cohorts with 20 or fewer individuals in subgroup analyses were not included in the meta-analysis. The average number of IBS patients per study was 192 (range 34–565).

**Fig. 2:**
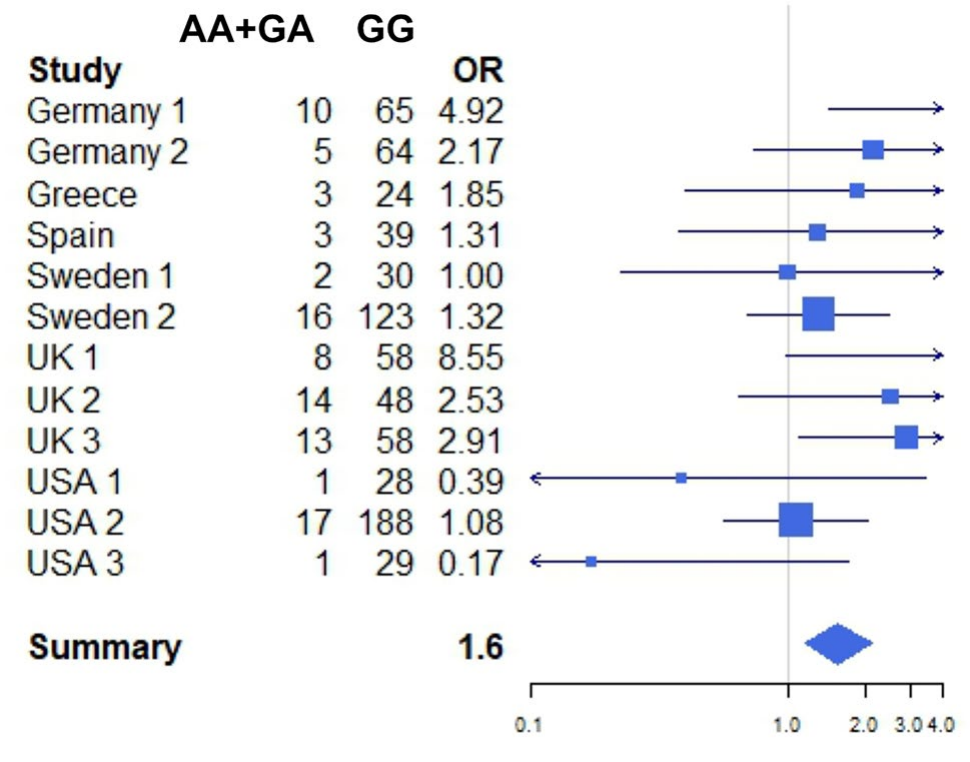
Forest-plots illustrating genotype relative risks of IBS phenotypes were quantified by odds ratios with corresponding 95% confidence intervals (indicated by lower and upper limit) based on a logistic regression model under dominant genetic penetrance for all but *HTR3C*. We assumed that the identified studies were random samples from a general population, and therefore used a random effects model to summarize odds ratio estimates in the meta-analyses of the respective gene SNP. Confidence intervals for each individual study were indicated by horizontal lines, single ORs by squares that reflected study sizes, and summary estimates by diamonds with horizontal limits at confidence limits and width inversely proportional to the standard error

**Fig. 3:**
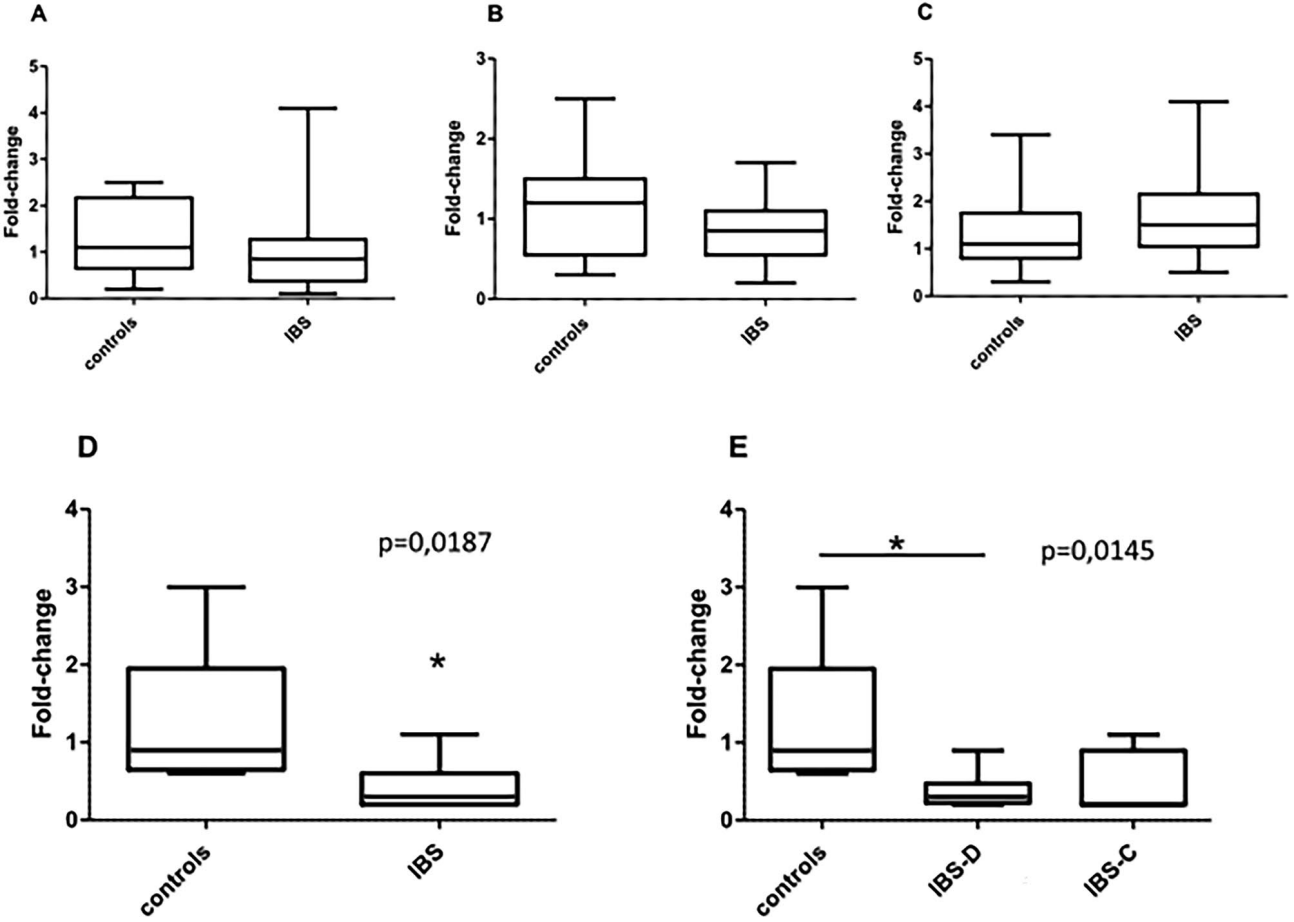
qPCR analysis of total RNA isolated from various GI regions including **A** jejunum, **B** ileum, **C** colon, and **D**, **E** sigmoid colon. Samples were run in triplicate and data analyzed by the 2-∆∆Ct method with correction for primer efficiency. Two-tailed parametric tests were used as appropriate (unpaired *t* test, one-way ANOVA followed by Bonferroni correction post-hoc test) using GraphPad Prism 5.0 software

Subsequent meta-analysis combining the initial datasets, that are the discovery sample from ‘UK1’ and the first replication sample ‘Germany 1’ [14, 15] with the replication data generated in our actual study only confirmed *HTR3E* c.*76G > A (rs56109847 = rs62625044) to be associated with female IBS-D (Mantel–Haenszel OR = 1.58; 95% CI (1.18,2.12)) (Table 1, Fig. 2, Supplementary SD Fig. 2). *HTR3E* genotypes did not deviate from HWE except for patients from the USA3 sample and the female controls in the Germany1 sample (see Supplementary SD Table 6). None of the other *HTR3* SNPs could be replicated (for further details see Supplementary SD Fig. 3).

To get an idea on the potential functional impact of the analyzed SNPs on differential expression regulation, we furthermore performed comparative expression analysis in samples from four different GI regions of 66 IBS patients and 42 controls (see Supplementary SD Table 2). In addition, we genotyped the respective polymorphisms *HTR3A* c.-42C > T (rs1062613), *HTR3B* p.129YS (rs1176744), *HTR3C* p.N163K (rs6766410), and *HTR3E* c.*76G > A (rs56109847 = rs62625044) in DNAs isolated from these GI tissues (jejunum, ileum, sigmoid colon, and colon). We furthermore aimed to compare expression levels of both, IBS patients and controls, and subsequently correlate expression levels with the genotype status. This analysis revealed that *HTR3E* was the only gene that was robustly expressed within all analyzed GI subregions (Ct value mostly below 30, see Supplementary SD Table 7). All other *HTR3* genes were not adequately expressed for further analysis (Ct value mainly above 30 or undetermined, see Supplementary SD Table 7).

Since the *HTR3E* SNP presents with a rather low minor allele frequency, not enough SNP carriers were available for correlation with expression levels. Of note, we found *HTR3E* transcript levels to be significantly reduced in the sigmoid colon of IBS patients, more specifically, in IBS-D patients (Fig. 3).

## Discussion

Various studies implicated the serotonin system and serotonin type 3 receptor genes, in particular, in the pathophysiology of IBS. We aimed to validate these findings in a large multi-center endeavor. However, the only SNP holding true to being associated with IBS was the *HTR3E* SNP c.*76G > A (rs56109847 = rs62625044) in females with IBS-D.

The lack of replication of the SNPs in *HTR3A*, *HTR3B*, and *HTR3C* with IBS may be attributed to an initial association with comorbid conditions like psychiatric disorders including depression and anxiety as well as pain disorders including migraine and fibromyalgia. Only recently, IBS was termed as gut-brain disorder and its comorbid conditions increasingly recognized. Accumulating evidence suggested an involvement of disturbed brain-gut axis communication. In fact, *HTR3A* rs1062613 had initially been associated with conditions seen in IBS-like depression and anxiety [27, 28], and had also been correlated with the severity of IBS symptoms and anxiety. It has also been linked to amygdaloid activity [29, 30], early life trauma and altered emotional networks in the human brain as well as with the onset of depression [31]. More recently, this SNP - in interaction with childhood trauma - was shown to differentially modulate central serotonin activity [32]. Lately, this SNP has been reported to contribute to pain intensity in temporomandibular disorder myalgia [33]. Furthermore, the HTR3B variant p.Tyr129Ser (rs1176744) has also been associated with bipolar affective disorder and female major depression as well as pain catastrophizing, a coping style characterized by excessively negative thoughts and emotions related to pain [22, 34–36]. Moreover, it has been associated with IBS, increased anxiety and alexithymia (a personality trait characterized by the inability to identify and describe emotions) [24]. All HTR3 SNPs have been proven 317 to be functional in earlier studies [12].

Interestingly, rs1062613 in *HTR3A* has also been associated with dyspepsia [37] and hypersensitivity in gastroesophageal reflux disease, possibly due to reduced 5-HT_3_ receptor activity in the descending serotonergic pathway [38]. Furthermore, in a functional imaging study of responses to rectal balloon distension, risk allele carriers presented with significantly more activation in the right amygdala, left insula, and left orbitofrontal cortex, suggesting that individuals carrying *HTR3* polymorphisms may respond differently to gut-derived signals in brain regions of negative emotion, body recognition, and that discrimination of the stimulus that might be enhanced in 5-HT_3_ receptor signaling [24].

In summary, disturbed peripheral and central 5-HT-mediated signaling seems to shape the phenotypes of these complex conditions. In line with this, functional MRI studies have confirmed that 5-HT_3_ receptors are important for the neural brain networks involved in emotional processing, learning, cognition, visceral perception, and pain processing. *HTR3* variants have been linked to functional and structural differences in brain regions relevant to these traits such as the amygdala, frontal cortex, temporal lobe, and insula [25, 30]. Therefore, an individual make-up of 5-HT3 receptors may specifically modulate relevant neural circuits, thereby making individuals prone to develop one or another disorder. The association findings of HTR3 variants with various complex disorders may additionally be attributed to a common genetic basis of neurodevelopmental disorders. 5-HT3 receptors are involved in shaping neuronal structure and plasticity in distinct brain regions during development and maturation [39]. HTR3-associated disorders may share neurodevelopmental and functional CNS and/or ENS disturbances, which may be altered by HTR3 variants. Modified responses may be distorted by disturbed 5-HT3 receptor signalling.

To understand the underlying molecular pathomechanisms and to allow the dissection of the respective disturbances that predispose to particular traits, deep phenotyping of patients and control individuals is mandatory.

In our qPCR analyses, none of the *HTR3* genes except *HTR3E* was found to be robustly expressed in any of the tested GI tissues from the small and large intestine. In contrast to our data, *HTR3A* was found to be robustly expressed in various brain and GI tissues in the GTex data set (Supplementary SD Fig. 4, access 16.12. 2020). This discrepancy can currently not yet be explained; however, but might be biased by inflammation in a given tissue. According to single cell data in the protein atlas, the cellular expression within the colon differs remarkably [41]. Whereas *HTR3E* was mainly found to be expressed in enteroendocrine and Paneth cells as well as in two different types of enterocytes (https://www.proteinatlas.org/ENSG00000186038-HTR3E/celltype/colon), the largest extent of *HTR3A* expression was mainly found in B cells of the colon, but only marginally in T cells as well as in another type of enterocytes as compared to *HTR3E* (https://www.proteinatlas.org/ENSG00000166736-HTR3A/celltype/colon).

The subordinate role of *HTR3B* and *HTR3C* within the GI tract is furthermore underlined by expression data from the GTEx portal (https://gtexportal.org/home/). This data shows the highest expression in the small and large intestines (terminal ileum data from *n* = 187 donors, median TPM (transcripts per million) = 0.4364, colon transverse data from *n* = 406 donors, median TPM (transcripts per million) = 0.4273). This is in line with earlier studies indicating that *HTR3E* is restricted to the GI tract, colon and ileum, respectively [42] (Supplementary SD Fig. 5, GTex access 16.12. 2020).

The expression in other tissues was even lower (e.g., brain cortex, *n* = 255 donors, median TPM = 0.019, all others: median TPM below 0.000).

Variants in *HTR3* genes might also alter the drug response. For example, in a recent pharmacogenetic study, stool consistency response to treatment with the 5-HT_3_ receptor antagonist ondansetron correlated with the CC genotype of the SNP p.N163K rs6766410 of the *HTR3C* gene [43] in IBS-D patients. To what extent *HTR3* SNPs might further influence drug response and therefore be relevant to novel pharmacogenetic treatments, will be the subject to future studies.

We are well aware that our study has some limitations. Methodological variations exist since we combined data from 14 centers from different countries with some samples of rather small size. However, to address the impact of the study size on the outcome of the meta-analysis, funnel plots were generated. Funnel plots of estimated log-ORs against their precision (1 divided by the standard error of the log-ORs) for each study were fairly symmetric around the overall effect estimate, i.e., no publication bias was evident (see Supplementary SD Fig. 6).

The number of tested SNPs was limited, and therefore we could not correct our data for population stratification using genetic principal component analysis. To mitigate potential bias, we restricted our analysis to individuals of European ancestry only. Of note, the replicated association of the *HTR3E* SNP seems to also hold true for the Asian population since four earlier studies confirmed this finding in case control studies from China [18–21].

How far our association finding may apply to other populations remains to be evaluated in future studies. Remarkably, some SNP genotype frequencies for *HTR3B* and *HTR3C* significantly deviated from the HWE expectation in patient subgroups from the German, Greek, US, and UK cohorts—a phenomenon which we cannot explain at present (see Supplementary SD Table 6). In addition, the *HTR3A* SNP data deviated from HWE in controls from Greece. *HTR3E* SNP data were in accordance with HWE for all cohorts but one US patient sample (IBS overall) and one German control sample (female controls). However, the estimated effect size and direction for the *HTR3E* SNP are in line with the overall results.

Another limitation relates to the definition of the IBS phenotype. We used Rome II and/or Rome III criteria and therefore could not refer to a uniform symptom classification. We are aware that, for example, the Rome III criteria [44] allow a wider diagnostic range of IBS, especially for IBS subtypes. To explore differences between ROME II- and ROME III-based diagnoses, a subgroup analysis was performed. Stratified analyses according to Rome II or Rome III criteria generally revealed stronger associations for Rome III diagnoses: IBS overall OR = 1.10, 95%CI (0.86–1.40) for Rome II compared to OR = 2.45, 95%CI (1.79–3.34) for Rome III; IBS-D OR = 1.31, 95%CI (0.96–1.80) for Rome II compared to OR = 2.23, 95%CI (1.51–3.27) for Rome III; IBS-D females OR = 1.57, 95%CI (1.07–2.29) for Rome II compared to OR = 1.94, 95%CI (1.16–3.22) for Rome III.

We are also facing a sex bias, since more females than males were included. Yet, this phenomenon might just reflect the fact that the prevalence of IBS is higher in women.

Moreover, we cannot exclude that IBS patients are included in the control samples since GI symptomatology that might qualify as IBS was not rigorously excluded in the control population. For genetic studies, a purely symptom-based IBS classification is only of limited specificity when it comes the identification of mechanistically diverse phenotypes of IBS or its subgroups [11]. Consequently, the assessment of additional symptoms as well as intermediate or quantitative traits, are all warranted to dissect the genetics underlying IBS subtypes and to correlate these to symptoms/biomarkers, applying a standardized and robust deep phenotypic tool [40]. Accounting for variations in the GI phenotype and also factoring the impact of common comorbidities and psychiatric phenotypes, personality traits, and somatization, in particular, should be a mandatory feature of future studies [40]. Of course, control individuals should be appropriately characterized to exclude IBS sufferers. Most of our samples came from tertiary referral centers, and our findings may therefore not hold true for all IBS patients.

The small number of samples in the differential expression analysis was another limitation. However, a strength of our study is the fact that we did not limit our study to only one GI region but instead, we included samples from the small and large intestines, whereas previous studies were limited to only one or two regions of the GI tract [45]. In addition, combined genotyping and expression analysis from the same individual was not possible due to the current design and should thus be a target in future studies.

In conclusion, meta-analysis confirmed the role of the *HTR3E* SNP rs56109847 = rs62625044 in females with IBS-D. Expression analysis revealed reduced *HTR3E* levels in the sigmoid colon of IBS-D patients. This underlines the relevance of *HTR3E* in the pathogenesis of IBS-D. To what extent this and the other non-replicated SNPs shape the GI phenotype in IBS and how they interfere with other behavior- and pain-related genetic variants and impact on communications via the gut-brain axis, remains currently unknown. Future studies are warranted to assess how these variants correlate with behavior, pain perception, and bowel habits and how far gene–gene and gene–environment interactions affect the individual susceptibility to chronic GI disorders. Our currently ongoing studies within the international H2020 consortium DISCOvERIE (*Development*, *dIagnosis and prevention of gender*-*related Somatic and mental COmorbiditiEs in iRritable Bowel Syndrome In Europe*, www.DISCOvERIE.eu) that implemented deep phenotyping guidelines [40] will allow us to get assess how *HTR3* variants relate to central and peripheral phenotypes.

A better understanding of the contribution of *HTR3* SNPs in shaping different phenotypes relevant to IBS and its comorbidities is of clinical importance and may allow insights into IBS pathophysiology and refine the targeting of 5-HT_3_ receptors in therapeutic interventions.

## Supplementary Information

Below is the link to the electronic supplementary material.

Supplementary file1 (XLSX 42 KB)

Supplementary file2 (DOCX 814 KB)

## Data Availability

Data will be available from the corresponding author upon reasonable request.

## Abbreviations

GI: Gastrointestinal
HWE: Hardy-Weinberg equilibrium
IBS: Irritable bowel syndrome
IBS-A: Alternating IBS
IBS-C: Constipation predominant IBS
IBS-D: Diarrhea predominant IBS
IBS-M: Mixed IBS
IBS-U: Unspecified IBS
5-HT3: Serotonin type 3 receptor
*HTR3*: Serotonin type 3 receptor gene
