# Supplementary material for: The serotonin receptor 3E variant is a risk factor for female IBS-D
Source: J Mol Med (Berl). 2022 Sep 19;100(11):1617–27. doi: 10.1007/s00109-022-02244-w (PMC9592668; doi:10.1007/s00109-022-02244-w)
Supplement: Supplementary file 2 — Supplementary file2 (DOCX 814 KB) [file 109_2022_2244_MOESM2_ESM.docx]

#### Supplementary Data

**Serotonin receptor type 3E variant is a risk factor for female IBS-D**

Nikola Fritz^1^, Sabrina Berens^2^, Yuanjun Dong^1^, Cristina Martínez^1,3,4^, Stefanie Schmitteckert^1^, Lesley A. Houghton^5,6^, Miriam Goebel-Stengel^7,8^, Verena Wahl^1^, Maria Kabisch^9^, Dorothea Götze^1^, Mauro D’Amato^10,11,12^, Tenghao Zheng^10^, Ralph Röth^1,13^, Hubert Mönnikes^14^, Jonas Tesarz^2^, Felicitas Engel^2^, Annika Gauss^15^, Martin Raithel^16^,Viola Andresen^17^, Jutta Keller^17^, Thomas Frieling^18^, Christian Pehl^19^, Christoph Stein-Thöringer^20^, Gerard Clarke^21,22^, Paul J. Kennedy^21,22^, John F. Cryan^21,22,23^, Timothy G. Dinan^21,22^, Eamonn M. M. Quigley^22,24^, Robin Spiller^25^, Caroll Beltrán^26^, Ana María Madrid^26^, Verónica Torres^26^, Emeran A. Mayer^27^, Gregory Sayuk^28^, Maria Gazouli^29^, George Karamanolis^30^, Mariona Bustamante^31,32^, Xavier Estivil^33^, Raquel Rabionet^33^, Per Hoffmann^34^, Markus M. Nöthen^34^, Stefanie Heilmann-Heimbach^34^, Börge Schmidt^35^, André Franke^36^, Wolfgang Lieb^37^, Wolfgang Herzog^2^, Guy Boeckxstaens^38^, Mira M. Wouters^38^, Magnus Simrén^39^, Gudrun A. Rappold^1,40^, Maria Vicario^41,42^, Javier Santos^41^, Rainer Schaefert^43,44^, Justo Lorenzo-Bermejo^9^, Beate Niesler^1,13,40^

^1^Institute of Human Genetics, Department of Human Molecular Genetics, Heidelberg University Hospital, Heidelberg, Germany

^2^Department of General Internal Medicine and Psychosomatics, Heidelberg University Hospital, Heidelberg, Germany

^3^Institut de Recerca Biomèdica de Lleida (IRBLleida), Lleida, Spain

^4^Lleida Institute for Biomedical Research Dr. Pifarré Foundation (IRBLleida), Lleida, Spain

^5^University of Leeds, St. James’s University Hospital, Leeds, UK

^6^Mayo Clinic, Jacksonville, FL, USA

^7^Department of Psychosomatic Medicine, University Hospital Tübingen, Tübingen, Germany

^8^Department of Internal Medicine and Gastroenterology, HELIOS Clinic Rottweil, Rottweil, Germany

^9^Institute of Medical Biometry and Informatics, Heidelberg University, Heidelberg, Germany

^10^Unit of Clinical Epidemiology, Department of Medicine Solna, Karolinska Institutet, Stockholm, Sweden

^11^ Gastrointestinal Genetics Lab, CIC bioGUNE - BRTA, Derio, Spain

^12^IKERBASQUE, Basque Foundation for Science, Bilbao, Spain

^13^nCounter Core Facility, Department of Human Molecular Genetics, Heidelberg University Hospital, Heidelberg, Germany

^14^Martin-Luther-Hospital, Berlin, Germany

^15^Department of Gastroenterology, Infectious Diseases and Intoxications, Heidelberg University, Heidelberg, Germany

^16^University of Erlangen, Erlangen, Germany

^17^Israelitisches Krankenhaus, Hamburg, Germany

^18^Helios Klinik Krefeld, Krefeld, Germany

^19^Krankenhaus Vilsbiburg, Vilsbiburg, Germany,

^20^German Cancer Research Center, Heidelberg, Germany

^21^Department of Psychiatry and Neurobehavioral Science, University College Cork, Cork, Ireland

^22^APC Microbiome Ireland, University College Cork, Cork, Ireland

^23^Department of Anatomy and Neuroscience, University College Cork, Cork, Ireland

^24^Lynda K. and David M. Underwood Center for Digestive Disorders, Houston Methodist Hospital, Weill Cornell Medical College, Houston, TX, USA

^25^Nottingham Digestive Diseases Centre, University of Nottingham, Nottingham, UK

^26^Gastroenterology Unit, Hospital Clínico Universidad de Chile, Medicine Department, Universidad de Chile, Santiago de Chile, Chile

^27^Oppenheimer Center for Neurobiology of Stress, University of California, Los Angeles, CA, USA

^28^Washington University School of Medicine, St. Louis, MO, USA

^29^Laboratory of Biology, Medical School, National and Kapodistrian University of Athens, Athens, Greece

^30^Academic Department of Gastroenterology, Medical School, National and Kapodistrian University of Athens, "Laikon" General Hospital, Athens, Greece

^31^CRG, Centre for Genomic Regulation, Barcelona, Spain

^32^ISGlobal, Barcelona, Spain

^33^Department of Genetics, Microbiology and Statistics, Faculty of Biology, IBUB, Universitat de Barcelona; CIBERER, IRSJD, Barcelona, Spain

^34^Life and Brain Center, Bonn, Germany

^35^Institute for Medical Informatics, Biometry and Epidemiology, University Hospital of Essen, Essen, Germany

^36^Institute of Clinical Molecular Biology, Kiel, Germany

^37^Institute of Epidemiology, Kiel, Germany

^38^TARGID, University Hospital Leuven, Leuven, Belgium

^39^Institute of Medicine, University of Gothenburg, Gothenburg, Sweden

^40^Interdisciplinary Center for Neurosciences (IZN), Heidelberg University, Heidelberg, Germany

^41^Institut de Recerca Vall d’Hebron, Hospital Vall d'Hebron, Passeig de la Vall d'Hebron, Barcelona, Spain

^42^Nestlé Institute of Health Sciences, Nestlé Research, Société des Produits Nestlé S.A., Vers-chez-les-Blanc, Lausanne, Switzerland

^43^Department of Psychosomatic Medicine, Division of Theragnostics, University Hospital Basel, Basel, Switzerland

^44^Faculty of Medicine, University of Basel, Basel, Switzerland

**Corresponding author**: Prof. Dr. rer. nat. Beate Niesler, Genetics of Neurogastroenterologic Disorders, Institute of Human Genetics, Department of Human Molecular Genetics, Heidelberg University, Im Neuenheimer Feld 366, 69120 Heidelberg, Germany, Tel.+49-6221-5635274, e-mail: [beate.niesler@med.uni-heidelberg.de](mailto:beate.niesler@med.uni-heidelberg.de)

#### Patients

**GlaxoSmithKline Cohort**

Two independent cohorts of IBS patients and controls from the UK (termed UK3) and the USA/Canada (termed USA2) from GlaxoSmithKline, previously termed ‘Belgium samples’ [1, 2] were included in this study. Informed consent was obtained from all participants and local ethics committees approved the study protocol. The demographics and clinical characteristics of both cohorts have previously been reported [3]. The IBS patients were divided into subgroups based on the pre-dominant bowel habit according to the Rome II criteria. All patients confirmed Caucasian ethnicity [3].

**Controls**

**Germany: Heinz-Nixdorf Recall Study**

Genotypes of German control individuals were taken from the Heinz-Nixdorf Recall (HNR) Study (<https://www.uni-due.de/recall-studie/>). Imputation was performed using IMPUTEv2 and 1000 Phase 3 genomes were used as a reference. The version of September 2014 was used, which contains 46.262.911 variants (SNPs, INDELs, SVs), 2504 persons, no monomorphs, no singletons, no X chromosome, no PAR regions. The impute2 files were converted to Plink formats using the best-guess method, i.e. the genotype with the highest probability was chosen as the genotype. Only SNPs with an info score >=0.8 were used. Afterwards all double markers were removed. The files were merged to one file (HNR.*). HNR_all.* also contains the unimputed chromosomes 23, 24 and 25. Finally, data from 2.723 individuals (1.364 male; 1.359 female) and 578.355 SNPs were generated of which the *HTR3* SNPs was selected for analysis in our study.

**PopGen Health Study**

**PopGen Controls**

Further German control data were taken from the PopGen Controls (<https://www.epidemiologie.uni-kiel.de/node/119>). *HTR3* SNPs were extracted from PopGen data of 1.228 individuals. SNPs not genotyped in PopGen: genotypes of 13 SNPs were imputed into 1.228 PopGen individuals based on 2.577 reference individuals from 1000 Genomes Project phase 3. IMPUTEv2 was used to perform genotype imputation (default settings) based on resulting genotype probabilities exact genotypes were determined according to threshold rule 0.9 (calls with uncertainty greater than 0.1 were treated as missing and the rest was treated as hard calls).

**Spain: INMA—INfancia y Medio Ambiente**

Genotypes of further Spanish controls were taken from the INMA - INfancia y Medio Ambiente-(Environment and Childhood) Project. This is a network of birth cohorts in Spain, that aim to study the role of environmental pollutants in air, water and diet during pregnancy and early childhood in relation to child growth and development (<http://www.proyectoinma.org/>) [4]. The study has been approved by the Ethical Committee of each participating centre and written consent was obtained from participating parents. Children from the following sub-cohorts participate in this study: INMA Menorca, INMA Sabadell, and INMA Valencia.

**Sweden-SALT - Screening Across the Lifespan Twin Study**

Additional genotypes of Swedish controls (SALT- Screening Across the Lifespan Twin study) were selected from a published IBS GWAS study on a Swedish general population cohort [2], where 534 IBS cases based on Rome criteria and 4.932 asymptomatic controls were included in the analyses. In this study, IBS controls were defined, if they reported no bowel symptoms in the interview. We randomly chose 2.000 Swedish controls for following replication studies.

#### SD Methods

**Subgroup analysis**

As some of the patients were diagnosed according to ROMEII and others to ROMEIII criteria, a subgroup analysis was performed to examine differences on diagnoses. The cohorts UK1 and USA1 were excluded of the analysis, as no information about individually applied ROME criteria was available.

**Genotyping**

Genotyping of the Greek Cohort was performed as outlined in the following. DNA was extracted from venous blood stored at -80ºC by alkaline lysis method using Nucleospin Blood Kit (Macherey-Nigel, GmbH & Co. KG, Düren, Germany). For *HTR3A* c.-42C>T (rs1062613) PCR-RFLP was used as previously described [5]. For the *HTR3B* variant p.Y129S (rs1176744) allele-specific PCR was performed. Two different PCR reactions are performed with one or the other allele specific primer. The primers used, were a common reverse: 5’ CACTACCATCTCCTAATCAGCC 3’, and a forward for the A-allele: 5’ AACATAGGGAAGGTCAGGGA 3’ and for the C-allele: 5’ AACATAGGGAAGGTCAGGGC 3’. The 117 bp fragment was amplified using an initial denaturation step for 5 min at 94°C, and then by 35 amplification cycles of denaturation at 94°C for 45 s, annealing at 56°C for 45 s, extension at 72°C for 45 s, and a final extension step at 72°C for 5 min. For *HTR3C* p.N163K (rs6766410) the PCR-RFLP method was used as well, with these forward 5’ TTCCGGTCTCACTGCCTATAT 3’, and reverse 5’ ACCACTCAAATGTCTTCCCTG 3’ primers. The 182 bp fragment was amplified applying an initial denaturation step for 5 min at 94°C, and then by 35 amplification cycles of denaturation at 94°C for 45 s, annealing at 65°C for 45 s, extension at 72°C for 45 s, and a final extension step at 72°C for 5 min. The 205 bp PCR products were digested with *EcoRII*. The presence of the C-allele resulted in two fragments of 127 and 78 bp, whereas the GA-allele remains uncut at 205 bp. For *HTR3E* c.*76G>A (rs56109847=rs62625044) also PCR-RFLP was applied as previously described [6].

Genotyping of the SNPs *HTR3A* c.-42C>T (rs1062613), *HTR3C* p.N163K (rs6766410) and *HTR3E* c.*76G>A (rs56109847=rs62625044) of the Sweden2 sample was performed at the Karolinska Institute as outlined previously [2].

**Genotype data work pipeline controls**

**Spain: INfancia y Medio Ambiente**

One thousand and seventy-one children, whose parents reported to be white and to be born in Spain or in European countries, and that were not lost during the 4y follow-up, were selected for genotyping. Genome-wide genotyping was performed using the HumanOmni1-Quad v1.0 Beadchip (Illumina) at the Spanish National Genotyping Centre (CEGEN). Genotype calling was done using the GeneTrain2.0 algorithm based on HapMap clusters implemented in the GenomeStudio software (Illumina). PLINK was used for the genetic data quality control. We applied the following initial quality control thresholds: sample call rate>98% and/or LRR SD<0.3 (excluded: N=7: 4 from VAL and 2 from MEN, 0.7%). Then, sex, relatedness (excluded: one duplicated sample and the younger brother of two brother-pairs detected in INMA SAB cohort), heterozygosity and population stratification were checked. Genetic variants were filtered for SNP call rate>95%, MAF>1% and HWE p value>1.10E-6. The final genetic data set consisted of 1.061 subjects from INMA Sabadell (N=396), INMA Valencia (N=349) and INMA Menorca (N=316) and 817.131 QCed SNPs in b36 and + strand.

Imputation was performed with IMPUTEv2 using as a cosmopolitan reference panel from the 1000 GENOME project (rel March 2012). After imputation the database consisted of 39.346-413 SNPs (38.546.049 imputed and 800.364 genotyped).

**Sweden SALT**

The SALT individuals were genotyped by Illumina OmniExpress platform and basic quality control metrics were applied as following, at both SNP and sample levels. Briefly, SNPs with call rates <95% and/or Hardy–Weinberg equilibrium (HWE) *P*<10^−5^ were excluded. Samples were removed, if: 1) call rates<98%, 2) genotype-imputed sex doesn’t match phenotype reported sex, 3) with an extreme heterozygosity rate (out of 3*SD of mean heterozygosity rate), 4) related with other samples (PI-HAT>0.1875), the one with higher missing rate in a related pair were removed.

Genome-wide imputation was then performed with a two-step imputation workflow based on Shapeit2 for phasing and IMPUTEv2 for imputation[7], using 1000 genomes cosmopolitan population phase3 as a reference panel. Before imputation, all genotypes were aligned on the same strand with reference panel by a tool called “Genotype harmonizer” [8]. All SNPs, that failed in strand alignment were excluded from analyses. After imputation, we only kept SNPs with high imputation quality (info value of 0.8 and higher).

**Statistical analysis of qPCR data**

Two-tailed parametric tests were used as appropriate (unpaired *t*-test, one-way ANOVA followed by Bonferroni correction post-hoc test) using GraphPad Prism 5.0 software (GraphPad Software, Inc., La Jolla; California). A Mann-Whitney *U* test and an unpaired t-test with Welch's correction were applied, when data did not follow a normal distribution.

**SD Tables**

See Excel file

**SD Figures**

**
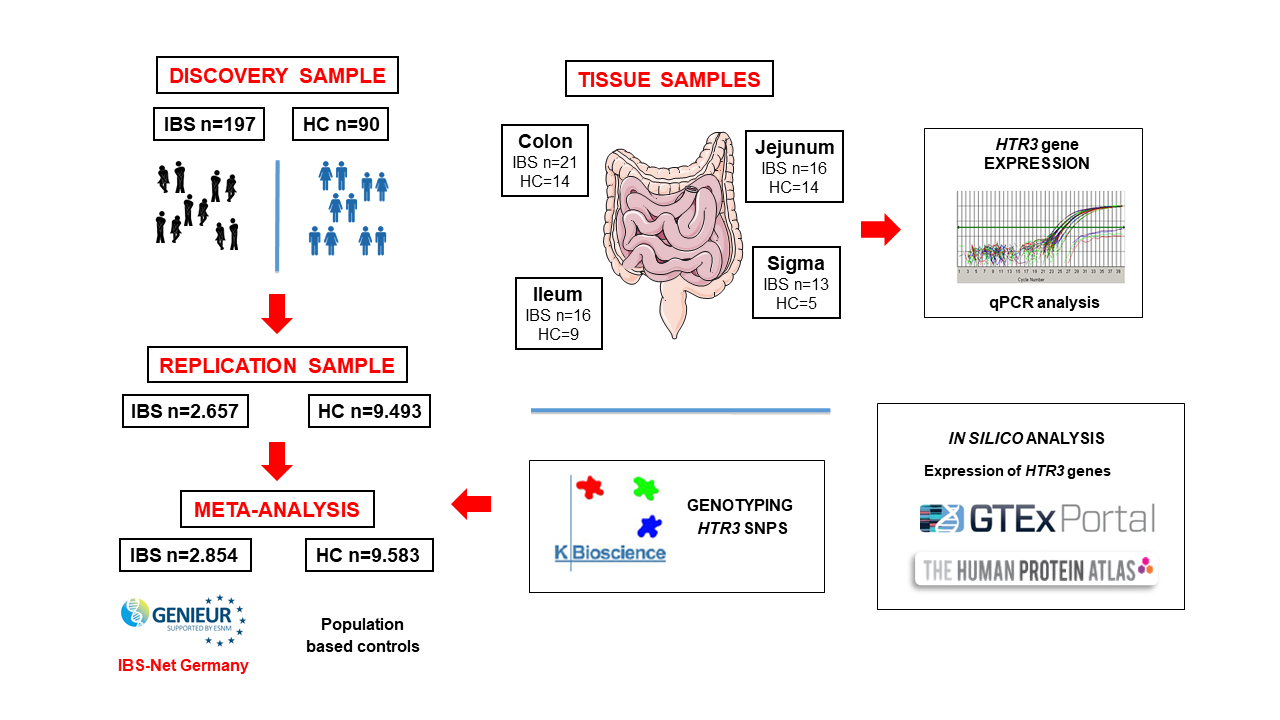
**

**SD Figure 1. Summary of experimental design.** Workflow of the analysis performed on biospecimens of human subjects and complementing *in silico* analyses into the functional relevance of detected variants. IBS - Irritable Bowel Syndrome, HC - healthy controls. (The figure was partly generated using Servier Medical Art, provided by Servier, licensed under a Creative Commons Attribution 3.0 unported license)


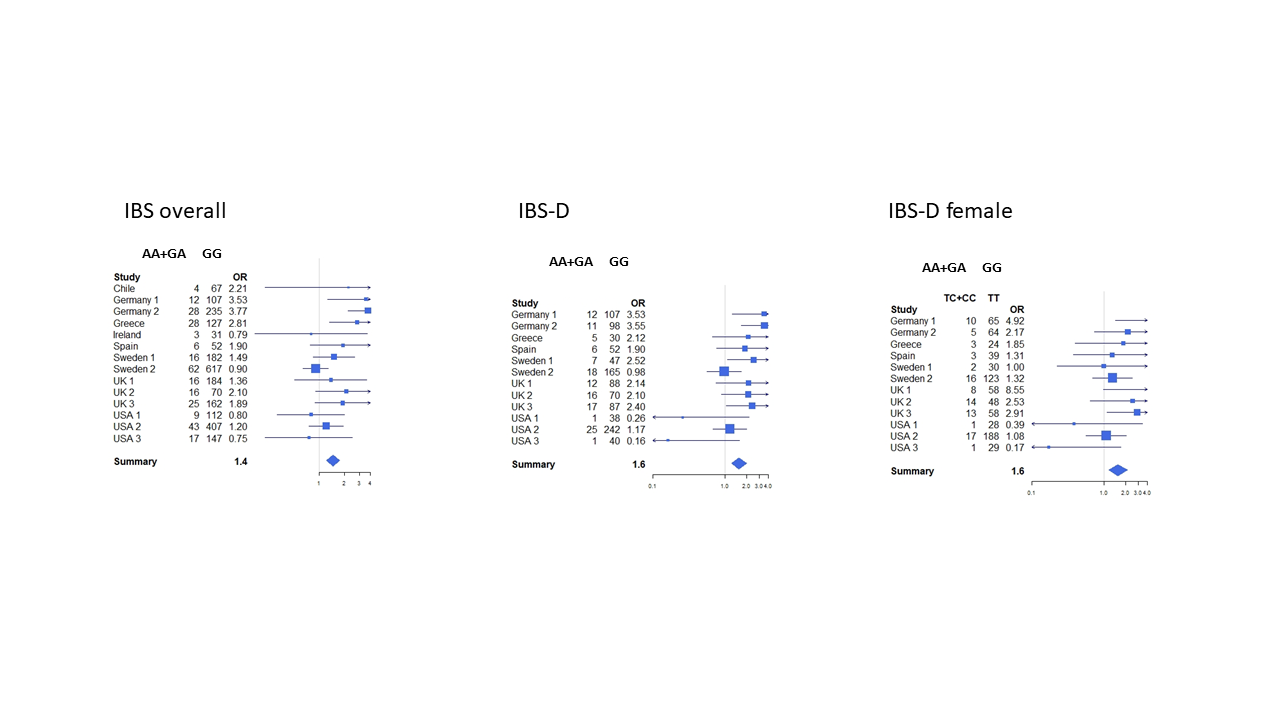


**SD Figure 2. Forest-plots illustrating genotype relative risks of IBS phenotypes: IBS overall, IBS-D and IBS-D female for the SNP *HTRE* c.*76G>A (rs56109847=rs62625044).** Data were quantified by odds ratios (ORs) with corresponding 95% confidence intervals (indicated by lower and upper limit) based on a logistic regression model under dominant genetic penetrance. We assumed, that the identified studies were random samples from a general population, and used a random effects model to summarize OR estimates in the meta-analyses of the respective gene SNP. Confidence intervals for each individual study are indicated by horizontal lines, single ORs by squares, that reflected study sizes. Summary estimates are displayed by diamonds with horizontal limits at confidence limits and width inversely proportional to the standard error.

**
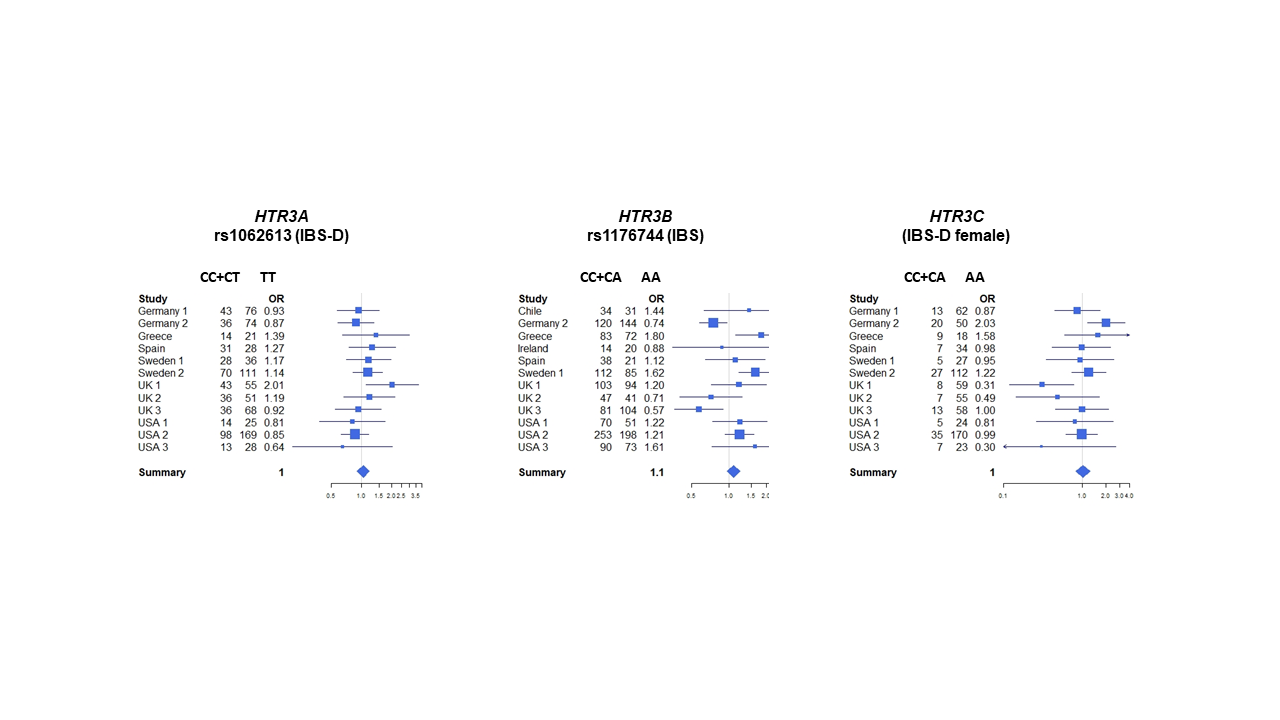
**

**SD Figure 3. Forest-plots illustrating genotype relative risks of IBS phenotypes.** Data were quantified by odds ratios (ORs) with corresponding 95% confidence intervals (indicated by lower and upper limit) based on a logistic regression model under dominant genetic penetrance for all but *HTR3C*. We assumed, that the identified studies were random samples from a general population, and used a random effects model to summarize OR estimates in the meta-analyses of the respective gene SNP. Confidence intervals for each individual study are indicated by horizontal lines, single ORs by squares, that reflected study sizes. Summary estimates are displayed by diamonds with horizontal limits at confidence limits and width inversely proportional to the standard error.


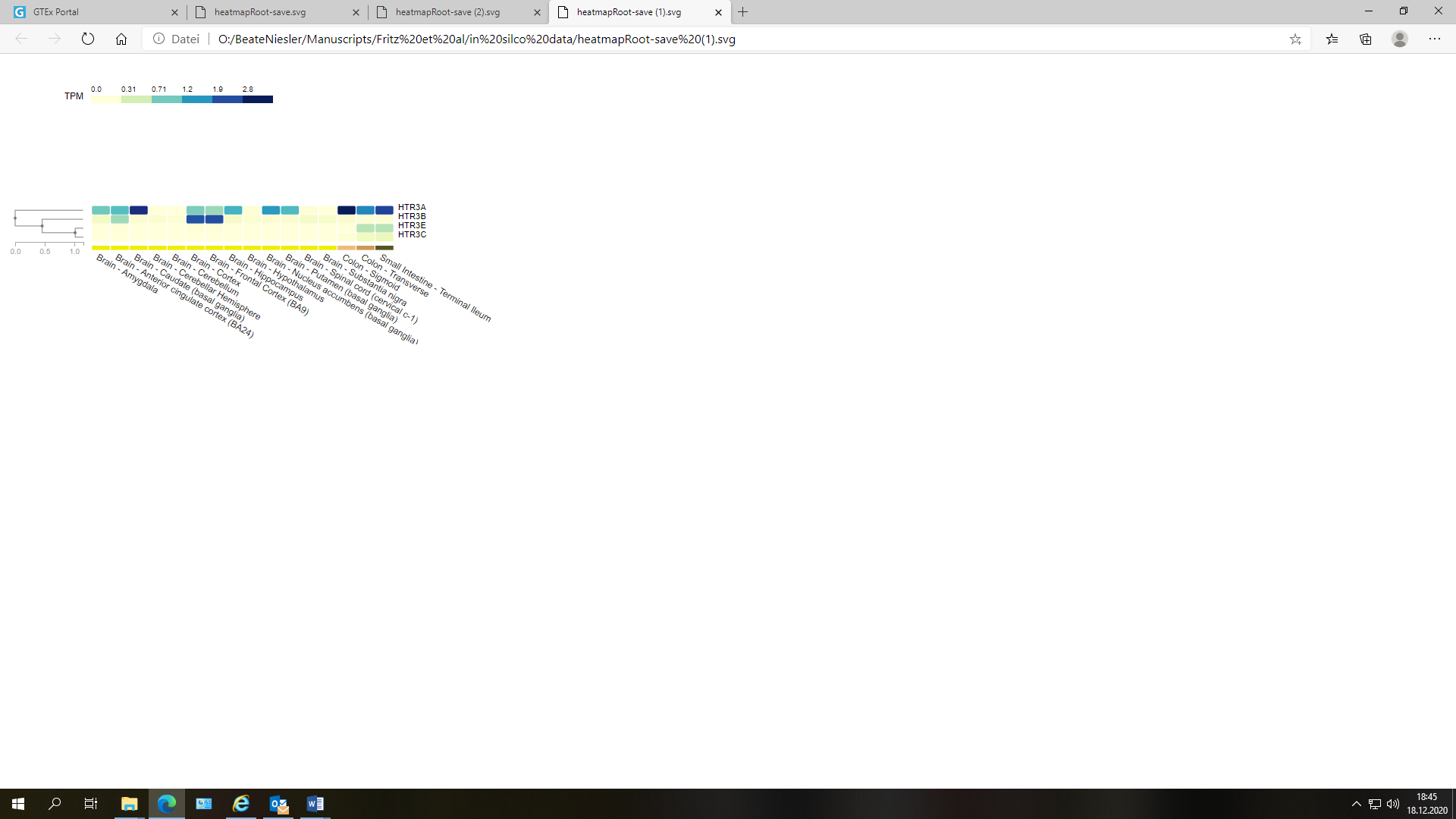


**SD Figure 4. *HTR3A, B, C and E* expression data** from brain and GI tissues from the GTEx portal (v7). *HTR3A* is most prominently and ubiquitously expressed, whereas the expression of *HTR3E* is restricted to the small and large intestines. In contrast, *HTR3B* appears to be confined to cortex and hippocampus in the brain. TPM (transcripts per million).


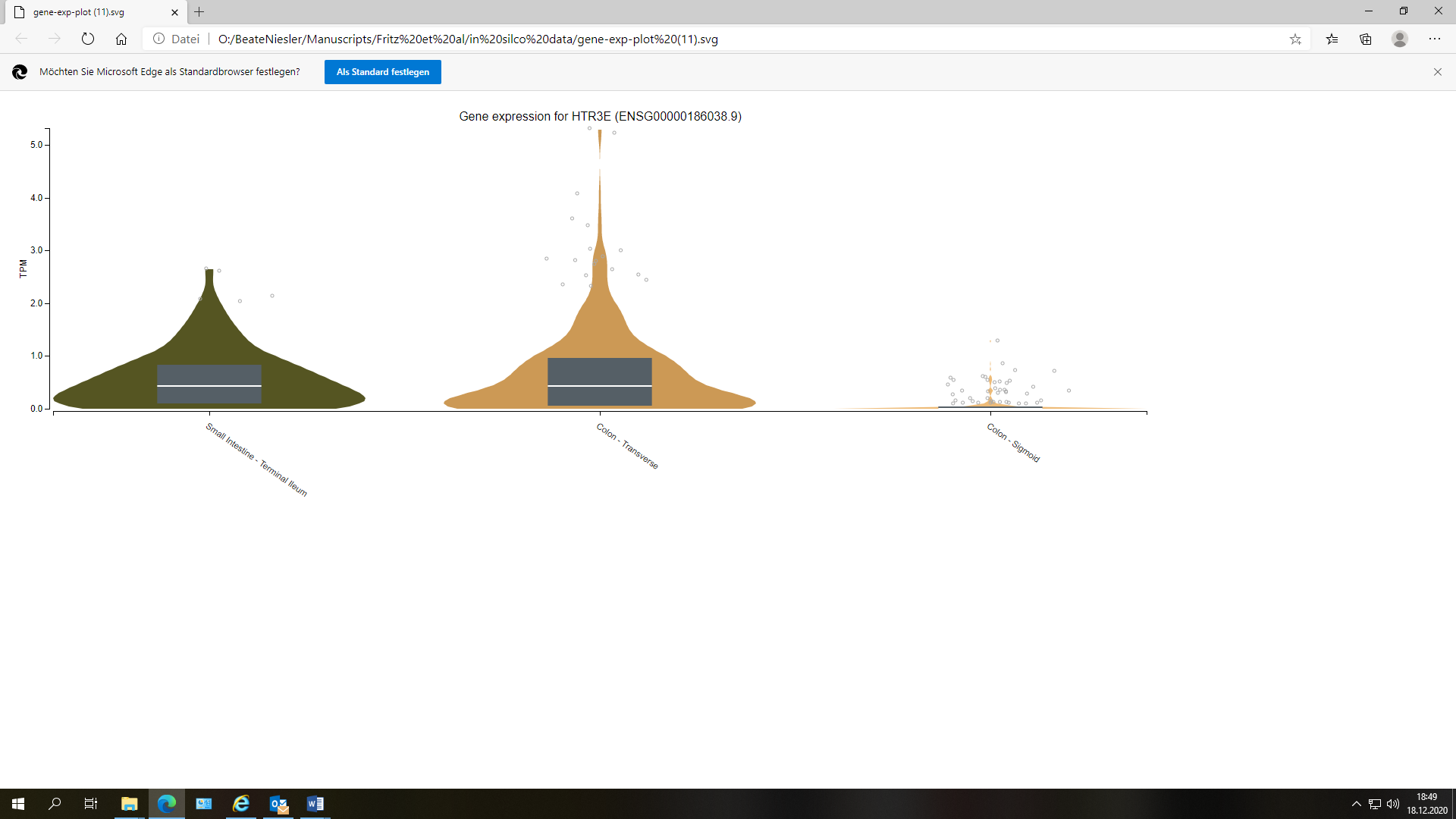


**SD Figure 5. *HTR3E* expression data from GI tissues** from the GTEx portal (v7). *HTR3E* is prominently expressed in the small and large intestines (terminal ileum data from n = 187 donors, median TPM (transcripts per million) = 0.4364, colon transverse data from n = 406 donors, median TPM (transcripts per million) = 0.4273).


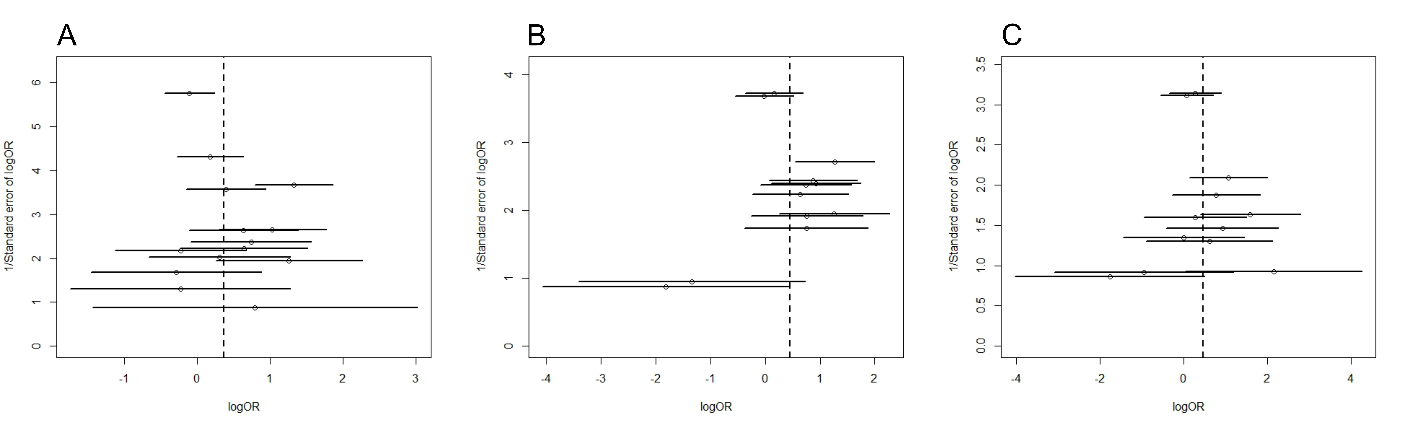


**SD Figure 6. Funnel plots** illustrating the impact of the study size on the outcome of the meta-analysis. Funnel plots of each study were fairly symmetric around the overall effect estimate. (A) IBS, (B) IBS-D, (C) IBS-D females.

**Acknowledgements**

We thank Dr. Theadore Ptak, Renee Henry, Ellen Goldstein, Cindy Lee, Deborah Roach, Jacqueline Rabuzin and Elizabeth Crosland (Toronto Digestive Disease Associates (TDDA Inc.), Toronto, Ontario, Canada), Dr. Mark Silverberg and Lori Baladjay (Mount Sinai Hospital, Toronto, Ontario, Canada), Dr. Yehuda Ringel, Dr. Robert Sandler, Alesia N. Aileo, Sarah Causey and Sarah Yeskel (University of North Carolina at Chapel Hill, NC, USA) as well as the staff at Ersta Hospital (Stockholm, Sweden) for collecting samples and acquiring data and Dr. Rachel Gibson and Dr. George Dukes (GlaxoSmithKline) for logistic support as well as for patient recruitment and clinical data collection.

Thanks to Milagros Gallart, Adoración Nieto, Laura Hernández and Sara Méndez from Hospital Universitari Vall d’Hebron for their invaluable assistance in the performance of jejunal biopsies. GB is supported by a grant from the Flanders Research Foundation (FWO, Odysseus). MW was supported by a FWO postdoctoral research fellowship. MD was funded by the Swedish Research Council (VR). APC Microbiome Ireland is funded by Science Foundation Ireland (SFI), through the Irish Government’s National Development Plan. The authors and their work were supported by SFI (grant number SFI/12/RC/2273 P2) and by the Health Research Board through Health Research Awards (grant no HRA_POR/2011/23; TGD, JFC, and GC). MS is supported by the Swedish Medical Research Council (grants 13409, 21691 and 21692), the Marianne and Marcus Wallenberg Foundation, and the University of Gothenburg, Centre for Person-Centred Care (GPCC), Sahlgrenska Academy, University of Gothenburg and by the Faculty of Medicine, University of Gothenburg; MV and JS are supported by Fondo de Investigación Sanitaria and CIBERehd, Instituto de Salud Carlos III, Subdirección General de Investigación Sanitaria, Ministerio de Economía y Competitividad (PI13/00935, PI14/00994); CM is supported by Instituto de Salud Carlos III, Subdirección General de Investigación Sanitaria, Ministerio de Ciencia, Innovación y Universidades (CP18/00116). The UK Nottingham cohort was supported by a Research for Patient Benefit grant and salary support for Dr. Garsed from the Nottingham Digestive Diseases Biomedical Research Unit. The Chilean Santiago cohort was supported by FONDECYT No11121527, No1181699.

The Genotype-Tissue Expression (GTEx) Project was supported by the [Common Fund](https://commonfund.nih.gov/GTEx) of the Office of the Director of the National Institutes of Health, as well as by NCI, NHGRI, NHLBI, NIDA, NIMH, and NINDS. Data described in this manuscript were obtained from the GTEx portal (Release v07 on 16 December 2020).

#### SD References

1. Wohlfarth C, Schmitteckert S, Hartle JD, Houghton LA, Dweep H, Fortea M, Assadi G, Braun A, Mederer T, Pohner S, et al. (2017) miR-16 and miR-103 impact 5-HT4 receptor signalling and correlate with symptom profile in irritable bowel syndrome. Sci Rep 7: 14680. DOI 10.1038/s41598-017-13982-0

2. Ek WE, Reznichenko A, Ripke S, Niesler B, Zucchelli M, Rivera NV, Schmidt PT, Pedersen NL, Magnusson P, Talley NJ, et al. (2015) Exploring the genetics of irritable bowel syndrome: a GWA study in the general population and replication in multinational case-control cohorts. Gut 64: 1774-1782. DOI 10.1136/gutjnl-2014-307997

3. Wouters MM, Lambrechts D, Knapp M, Cleynen I, Whorwell P, Agreus L, Dlugosz A, Schmidt PT, Halfvarson J, Simren M, et al. (2014) Genetic variants in CDC42 and NXPH1 as susceptibility factors for constipation and diarrhoea predominant irritable bowel syndrome. Gut 63: 1103-1111. DOI 10.1136/gutjnl-2013-304570

4. Guxens M, Ballester F, Espada M, Fernandez MF, Grimalt JO, Ibarluzea J, Olea N, Rebagliato M, Tardon A, Torrent M, et al. (2012) Cohort Profile: the INMA--INfancia y Medio Ambiente--(Environment and Childhood) Project. Int J Epidemiol 41: 930-940. DOI 10.1093/ije/dyr054

5. Gu QY, Zhang J, Feng YC, Dai GR, Du WP (2015) Association of genetic polymorphisms in HTR3A and HTR3E with diarrhea predominant irritable bowel syndrome. Int J Clin Exp Med 8: 4581-4585

6. Zhang Y, Li Y, Hao Z, Li X, Bo P, Gong W (2016) Association of the Serotonin Receptor 3E Gene as a Functional Variant in the MicroRNA-510 Target Site with Diarrhea Predominant Irritable Bowel Syndrome in Chinese Women. J Neurogastroenterol Motil 22: 272-281. DOI 10.5056/jnm15138

7. van Leeuwen EM, Kanterakis A, Deelen P, Kattenberg MV, Genome of the Netherlands C, Slagboom PE, de Bakker PI, Wijmenga C, Swertz MA, Boomsma DI, et al. (2015) Population-specific genotype imputations using minimac or IMPUTE2. Nat Protoc 10: 1285-1296. DOI 10.1038/nprot.2015.077

8. Deelen P, Bonder MJ, van der Velde KJ, Westra HJ, Winder E, Hendriksen D, Franke L, Swertz MA (2014) Genotype harmonizer: automatic strand alignment and format conversion for genotype data integration. BMC Res Notes 7: 901. DOI 10.1186/1756-0500-7-901
